# Screening for carotid atherosclerosis: development and validation of a high-precision risk scoring tool

**DOI:** 10.3389/fcvm.2024.1392752

**Published:** 2024-07-25

**Authors:** Zhi-Xin Huang, Lijuan Chen, Ping Chen, Yingyi Dai, Haike Lu, Yicheng Liang, Qingguo Ding, Piaonan Liang

**Affiliations:** ^1^ Department of Neurology, The Affiliated Guangdong Second Provincial General Hospital of Jinan University, Guangzhou, Guangdong, China; ^2^ Guangzhou University of Chinese Medicine, Guangzhou, Guangdong, China; ^3^ Department of Ultrasound, Songyang County People’s Hospital, Lishui, Zhejiang, China; ^4^ Department of Neurology, The First Hospital of Putian City, Putian, Fujiang, China; ^5^ Department of Neurology, Nanhai Economic Development Zone Peoples Hospital, Foshan, Guangdong, China; ^6^ Department of Rehabilitation Medicine, The Affiliated Guangdong Second Provincial General Hospital of Jinan University, Guangzhou, Guangdong, China

**Keywords:** prevention, cerebrovascular disease, screening, ultrasound, machine learning, carotid atherosclerosis

## Abstract

**Objective:**

This study aimed to investigate the prevalence of carotid atherosclerosis (CAS), especially among seniors, and develop a precise risk assessment tool to facilitate screening and early intervention for high-risk individuals.

**Methods:**

A comprehensive approach was employed, integrating traditional epidemiological methods with advanced machine learning techniques, including support vector machines, XGBoost, decision trees, random forests, and logistic regression.

**Results:**

Among 1,515 participants, CAS prevalence reached 57.4%, concentrated within older individuals. Positive correlations were identified with age, systolic blood pressure, a history of hypertension, male gender, and total cholesterol. High-density lipoprotein (HDL) emerged as a protective factor against CAS, with total cholesterol and HDL levels proving significant predictors.

**Conclusions:**

This research illuminates the risk factors linked to CAS and introduces a validated risk scoring tool, highlighted by the logistic classifier's consistent performance during training and testing. This tool shows potential for pinpointing high-risk individuals in community health programs, streamlining screening and intervention by clinical physicians. By stressing the significance of managing cholesterol levels, especially HDL, our findings provide actionable insights for CAS prevention. Nonetheless, rigorous validation is paramount to guarantee its practicality and efficacy in real-world scenarios.

## Introduction

Carotid atherosclerosis (CAS) is not just an indication of systemic atherosclerosis in the carotid arteries; its implications extend far beyond that (1, 2). Firstly, CAS had garnered significant attention due to its close association with stroke (3), a grave health concern where CAS was recognized as a major contributing factor. Notably, asymptomatic CAS exhibits a high prevalence of up to 40% among middle-aged and elderly individuals, implying that it may have remained unnoticed in many individuals (4). Rupture of atherosclerotic plaques in the carotid arteries could trigger transient ischemic attacks and strokes, profoundly impacting patients’ lives. Moreover, given atherosclerosis was a systemic disease, there exists a close relationship between CAS and coronary atherosclerosis, further elevating the risk of cardiovascular and cerebrovascular diseases (5, 6). These multiple hazards underscored the urgent need for in-depth research and management of CAS.

In the process of studying and managing CAS, ultrasound diagnosis played a pivotal role (7). Carotid ultrasound examination provides crucial insights into the disease severity. It facilitated early diagnosis and monitoring of disease progression by quantifying carotid intima-media thickness (CIMT) and detecting carotid atherosclerotic plaques (CAP) (8, 9). This non-invasive tool boasted safety benefits without radiation risks while remaining applicable across extensive age groups from children to seniors. These advantages made ultrasound diagnosis a powerful methodology for elucidating the underpinnings and clinical approaches for CAS. Our focus was on the overlooked community populations. We utilized ultrasound techniques for CAS screening, aiming to ascertain subjects at risk and further evaluate associated factors. This endeavor provides valuable data that could enable the establishment of a robust scoring model to accurately evaluate CAS risks in patients and tailor preventive tactics. Through this study, we aim to thoroughly characterize and strategize management of CAS, especially in community settings, filling knowledge gaps to enhance screening and care.

## Methods

### Study population

The study protocol was reviewed and approved by the Ethical Committee of Guangdong Second Provincial General Hospital (Approval number: GD2H-KY IRB-AF-SC.07-01.2), and the ethical guidelines of the 1975 Declaration of Helsinki were followed.

A prospective longitudinal study named Ivy Action was performed in the form of a voluntary prevention screening program for ischemic cerebrovascular disease targeting the adult population of multicenter communities (Guangzhou) in 2018–2019. The study included individuals aged 35 years or older, who had no history of stroke or had experienced a good recovery after stroke with a modified Rankin scale score of 2 or less indicating good recovery. Exclusion criteria included individuals unable to communicate with the research team, those with mobility impairments hindering study participation, individuals with heart, liver, or kidney failure, and patients with a history of malignancy. The comprehensive study protocol included gathering information on basic socio-economic status, social and residential status, smoking, housework, physical activity, sleep and dietary habits. Additionally, cardiovascular risk factors, family history and medical history were meticulously recorded.

In our machine learning models, we employed a comprehensive set of risk factors, including age, gender, biomarkers, systolic and diastolic blood pressure, health status, lifestyle factors, family history of cardiovascular disease, and medical history (Table 1). These factors were chosen for their well-documented association with carotid atherosclerosis and their clinical significance in predicting cardiovascular events. The medical exam included non-invasive tests (resting blood pressure, anthropometric measurements), ECG, anxiety and depression Scale, venous blood tests performed in a central laboratory with conventional enzymatic methods, echocardiography and carotid duplex scans (10). Waist and hip circumference were measured with light clothing. Cardiac History encompasses any medical history of coronary artery disease, myocardial infarction, angina, atrial fibrillation, or valvular heart disease. A waist-to-hip ratio over 0.90 for men and >0.85 for women was considered central obesity.

**Table 1:** Demographic information for participants.

Characteristic	No, *N* = 646^a^	Yes, *N* = 869^a^
Sex		
Male	157 (24%)	350 (40%)
Female	489 (76%)	519 (60%)
Age	54 (45, 60)	63 (59, 68)
Biomarkers		
hsCRP	0.85 (0.43, 1.78)	1.14 (0.59, 2.39)
Unknown	2	5
Uric Acid	329 (274, 396)	351 (297, 428)
Unknown	2	2
Hemoglobin	5.60 (5.30, 5.80)	5.70 (5.50, 6.10)
Unknown	2	2
Glucose	4.69 (4.34, 5.18)	5.03 (4.58, 5.91)
Unknown	2	2
Homocysteine	10.4 (8.8, 12.6)	11.7 (9.8, 14.3)
Unknown	2	2
LDL	2.83 (2.37, 3.30)	3.00 (2.45, 3.63)
Unknown	2	2
TG	1.27 (0.93, 1.90)	1.50 (1.10, 2.08)
Unknown	2	2
HDL	1.31 (1.15, 1.52)	1.29 (1.13, 1.49)
Unknown	2	2
TC	5.26 (4.65, 5.91)	5.42 (4.74, 6.16)
Unknown	2	2
Blood pressure		
SBP	120 (109, 131)	133 (121, 146)
Unknown	18	11
DBP	79 (73, 86)	82 (75, 89)
Unknown	18	11
Medical history		
Hypertension	100 (15%)	330 (38%)
Dyslipidemia	116 (18%)	264 (30%)
Diabetes	29 (4.5%)	114 (13%)
Cardiac history	26 (4.0%)	109 (13%)
Depression or anxiety	124 (19%)	154 (18%)
Cognitive impairment	13 (2.0%)	19 (2.2%)
Family history		
Stroke	103 (16%)	140 (16%)
Unknown	0	1
Coronary heart disease	65 (10%)	105 (12%)
Unknown	0	1
Hypertension	245 (38%)	325 (37%)
Unknown	0	1
Diabetes	106 (16%)	109 (13%)
Unknown	0	1
Health status		
BMI	23.2 (21.5, 25.2)	23.8 (21.8, 25.8)
Unknown	16	14
Central obesity	323 (50%)	592 (68%)
Smoking	76 (12%)	159 (18%)
Unknown	1	2
Drinking	103 (16%)	204 (24%)
Unknown	2	3
Sleep	357 (56%)	492 (57%)
Unknown	10	6
Lifestyle factors		
Living alone	23 (3.6%)	44 (5.1%)
Unknown	1	3
Sport levels		
Lack of physical activity	270 (50%)	355 (47%)
Frequent physical activity	270 (50%)	393 (53%)
Unknown	106	121
Housework Level		
No	37 (6.3%)	73 (9.0%)
Occasionally	46 (7.8%)	68 (8.4%)
30 min_perday	35 (5.9%)	55 (6.8%)
30 min–1 h_perday	90 (15%)	130 (16%)
1–3 h_perday	243 (41%)	307 (38%)
4 h_perday	140 (24%)	180 (22%)
Unknown	55	56
Mutual action		
Virtually none	221 (39%)	299 (38%)
1–2days_perweek	214 (38%)	278 (35%)
3–5days_perweek	85 (15%)	138 (17%)
6–7days_perweek	46 (8.1%)	80 (10%)
Unknown	80	74

^a^ *n* (%); Median (IQR); “unknown” indicates missing values.

It is essential to note that the primary aim of this study was focused on the prevention and management of stroke. All examination procedures were provided free of charge, and participants had the option to voluntarily undergo testing, ensuring that the study adhered to ethical standards and obtained informed consent from the participants.

### Utilizing high-resolution ultrasound to assess CAS

An examination was conducted with the patient supine with the head slightly extended. Carotid intima media thickness (IMT) was evaluated with a 7.5 MHz linear array transducer using a LOGIO E ultrasound (GE healthcare, USA). Measurements were taken carefully, analyzing the maximum IMT at two sites and all interfaces of the near and far walls of the common carotid artery (CCA), located 1–1.5 cm below its bifurcation. To obtain anechoic images, adjustments the image gain, depth, and focus were meticulously made for each participant. Plaques were defined as a focal thickening extending into the lumen by at least 0.5 mm or as IMTs greater than 1.5 mm, measured between the near- and far-walls of any carotid segment in the internal carotid artery, common carotid arteries, and carotid bulb. Carotid artery (CA) was defined as having a maximal intima-media thickness (IMT) of ≥1.0 mm and/or the presence of atherosclerotic plaques. To minimize intra-operator variation, a single experienced sonographer conducted all ultrasound examinations.

### A comprehensive approach to machine learning modeling and evaluation

The analytical approach employed in this study leveraged R code to perform several critical steps. To address missing data, the “missRanger” package was utilized for imputing missing values. Subsequently, a diverse set of machine learning models, encompassing support vector machines (SVM), XGBoost, decision trees, random forests, and logistic regression, were employed. Hyperparameter tuning was conducted through random search to identify the optimal model configuration. To ensure robust model performance while mitigating overfitting, a 5-fold cross-validation strategy was implemented. Given the presence of imbalanced target classes, data balance was achieved through oversampling. Model performance evaluation primarily relied on the area under the curve (AUC) metric, facilitating the selection of the best-performing machine learning method as the final model.

In the final model evaluation, three key approaches were employed: (1) ROC and PRC (Precision-Recall Curve) plots provided in-depth insights into model performance. (2) Model calibration was conducted using the calibration function from the “calibrate” package. This process involved generating calibration plots for apparent and bias-corrected probabilities to comprehensively assess model performance. (3) Decision Curve Analysis (DCA) was carried out to evaluate the clinical utility of the model. Decision curves and the plot_decision_curve function were employed to visualize the net benefit of the model across various threshold probabilities.

Additionally, a forest plot was created using the forest_model function from the “forestmodel” package, offering deeper insights into the relationship between predictor variables and the outcome variable. Sample size calculation was executed using the ShowRegTable function, contributing to an understanding of dataset characteristics and ensuring an adequate sample size for the analysis.

In summary, this methodological approach comprised data imputation, machine learning modeling, hyperparameter tuning, cross-validation, data balancing, and comprehensive model performance evaluation, culminating in the selection of the best-performing model. The final model evaluation involved ROC and PRC plots, model calibration, Decision Curve Analysis, and a forest plot. Sample size calculations were conducted to support the robustness of the analysis.

### Developing a risk scoring system

Grouping and Reference Value Selection of Risk Factors: Firstly, risk factors are grouped based on their clinical significance or common usage. In each group, appropriate numerical values are selected as reference values (Wij). Typically, the midpoint of the group is chosen as the reference value. Secondly, Handling Categorical Variables: For categorical variables such as gender, a category is chosen as the reference, and its reference value is set to 0, while other categories are naturally assigned numerical values, typically 1. Basic Risk Reference Value: Each risk factor needs to have a suitable group selected as the basic risk reference value (WiREF). When constructing a scoring tool later, the score for this group will be set as 0. Scores for other groups will be assigned positive or negative values based on their relationship with WiREF. Scoring Calculation: Using the regression coefficients estimated by a multiple logistic regression model (*β*i) and the reference values for each risk factor group (Wij), the distance between each risk factor's group and the basic risk reference value (WiREF) is calculated (D). The calculation formula is D = (Wij−WiREF) * *β*i. Thirdly, Risk Probability Calculation: Using the equation of a multiple logistic regression model, the probability of risk prediction $\left(\hat{\;p}\right)$ for each score is calculated. This probability value represents the likelihood of an individual experiencing a specific event under certain risk factors. The formula is:

$$\hat{\;p}=\frac{1}{1+\exp\left(-\sum_{i=0}^{p}\beta iXi\right)},$$

where $\sum_{i=0}^{p}\beta iXi$ represents a linear combination, which is approximately a constant term added to the product of the scores for various risk factors. Analyses were conducted using the R Statistical language (version 4.3.1; R Core Team, 2023).

## Results

### Population characteristics

Initially, 1,560 participants were recruited; however, 45 individuals with incomplete carotid ultrasound examinations were excluded from analysis. Consequently, the final analytical sample comprised 1,515 subjects. Carotid ultrasound revealed CAS in 869 participants, constituting a prevalence of 57.4% in the study cohort. Individuals in the CAS group demonstrated more advanced age compared to the non-CAS group (mean 63 years vs. 54 years). Additionally, the majority of CAS individuals were female (519 subjects, accounting for prevalence of 60%). Detailed demographic information for the entire study population is presented in Table 1.

### Machine learning model training and evaluation process

**Figure 1:**
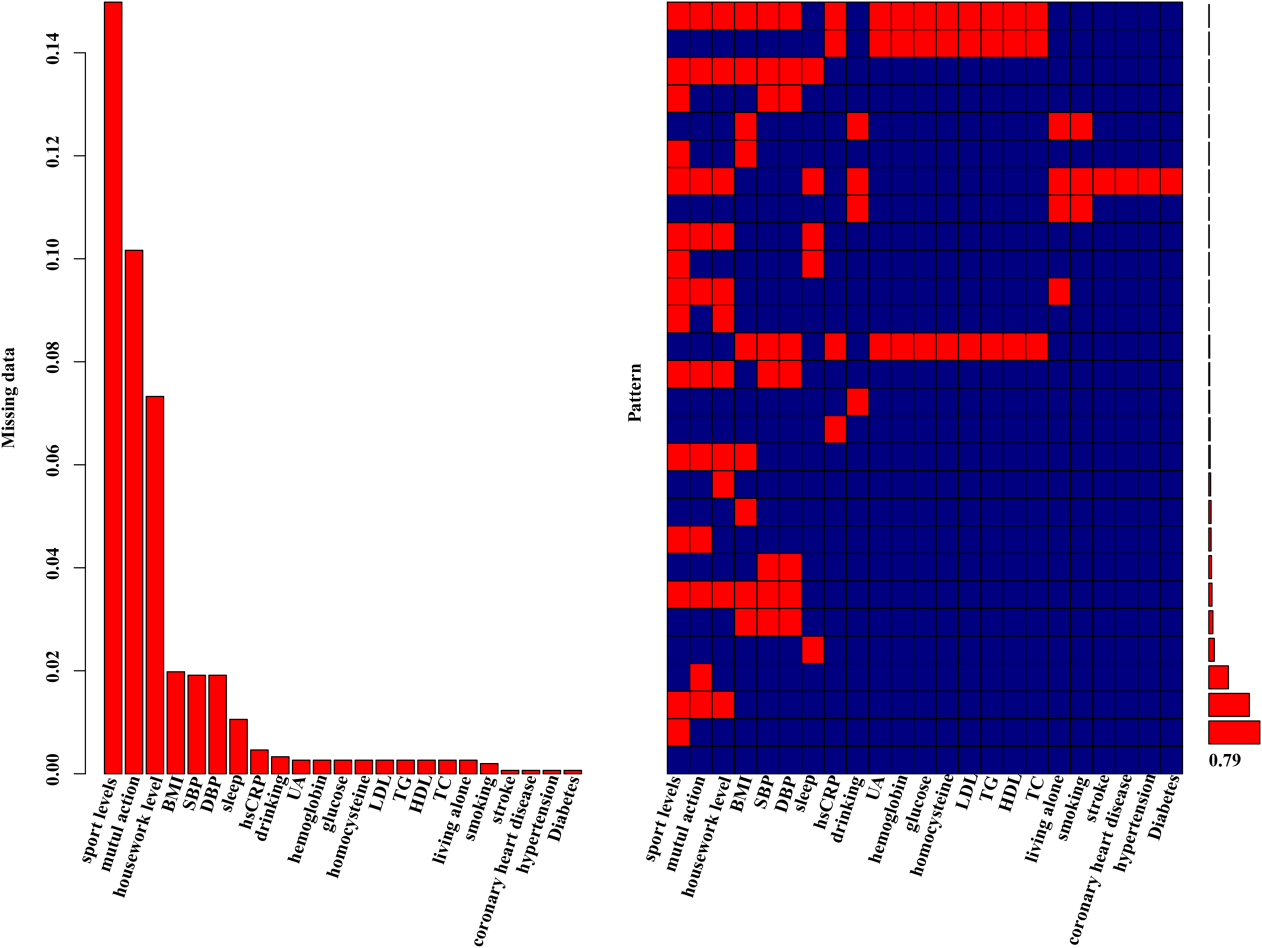
Missing data aggregation plot. On the left, the distribution of missing data is depicted as a percentage. On the right, a missing pattern analysis (aggregation missingness plot using the VIM package) is presented, showcasing the percentages of different missing patterns. 79% of the entire study population had no missing values.

**Figure 2:**
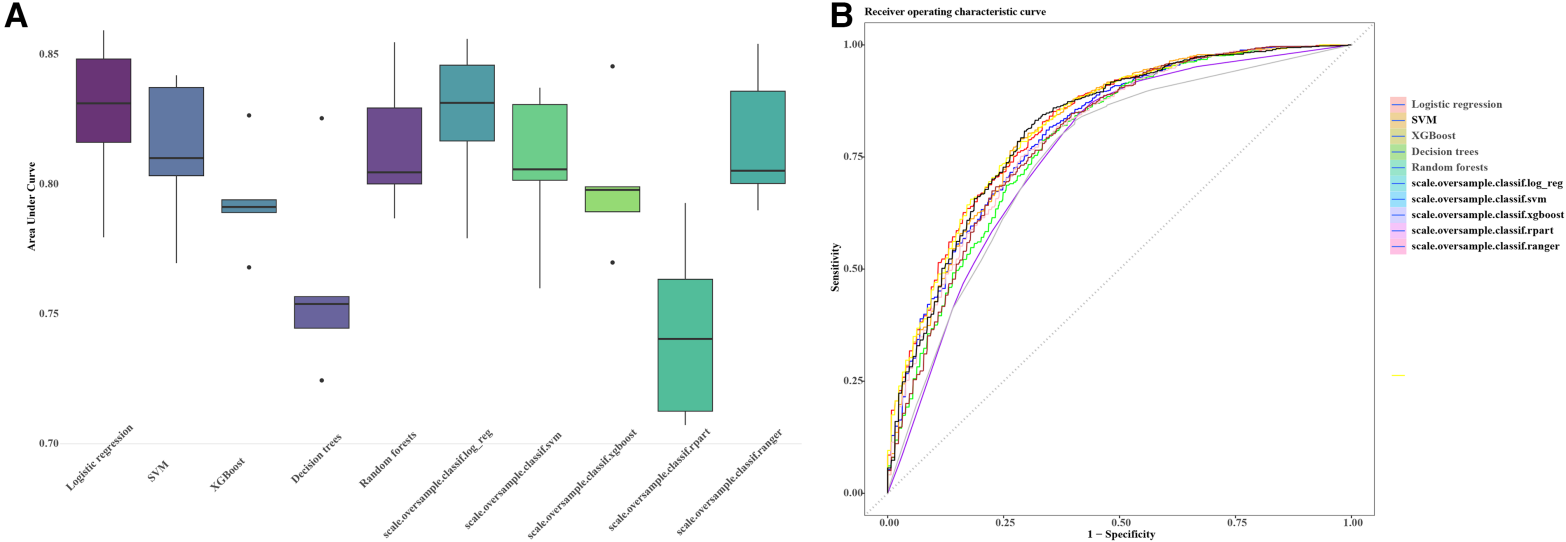
AUC and ROC comparisons for imbalanced classification learners. The figure illustrates the AUC (**A**) and ROC (**B**) comparisons for different learners in the context of imbalanced classification problems. Each learner is represented by a distinct color. The AUC/ROC value, denoted in the legend, quantify the overall classification performance of each learner. The higher the AUC/ROC, the better the learner's ability to distinguish between classes. This set of machine learning pipelines (scale.oversample.classif.log_reg, scale.oversample.classif.svm, scale.oversample.classif.xgboost, scale.oversample.classif.rpart, scale.oversample.classif.ranger) is designed to tackle imbalanced classification problems. Firstly, by scaling features, the data is normalized to enhance the robustness of the classification algorithms. Secondly, to address class imbalance, oversampling techniques are applied to augment the number of samples in the minority class. Finally, each pipeline employs a different classifier, such as logistic regression, support vector machine, XGBoost, recursive partitioning trees (rpart), and Ranger random forest, to accomplish the ultimate classification task. This integrated framework aims to improve the model's ability to handle imbalanced data, thereby enhancing the overall performance of the classification algorithms. AUC, area under the curve; ROC, receiver operating characteristic curve.

The preprocessing phase involved imputing missing values to ensure a complete dataset, as depicted in Figure 1. Subsequently, five machine learning models were explored, including logistic regression, support vector machines (SVM), XGBoost, decision trees and random forests. Hyperparameter optimization was carried out through random search across defined parameter spaces for each algorithm. To mitigate class imbalance, the “oversample” method from the classbalancing package was utilized, equalizing the training set's target categories. For imbalanced classification, the area under the receiver operating characteristic curve (AUC) served as a robust metric of overall performance. As shown in Figure 2, the ROC curves described each learner's discrimination capacity across thresholds, with the AUC score quantifying overall predictive power.

**Figure 3:**
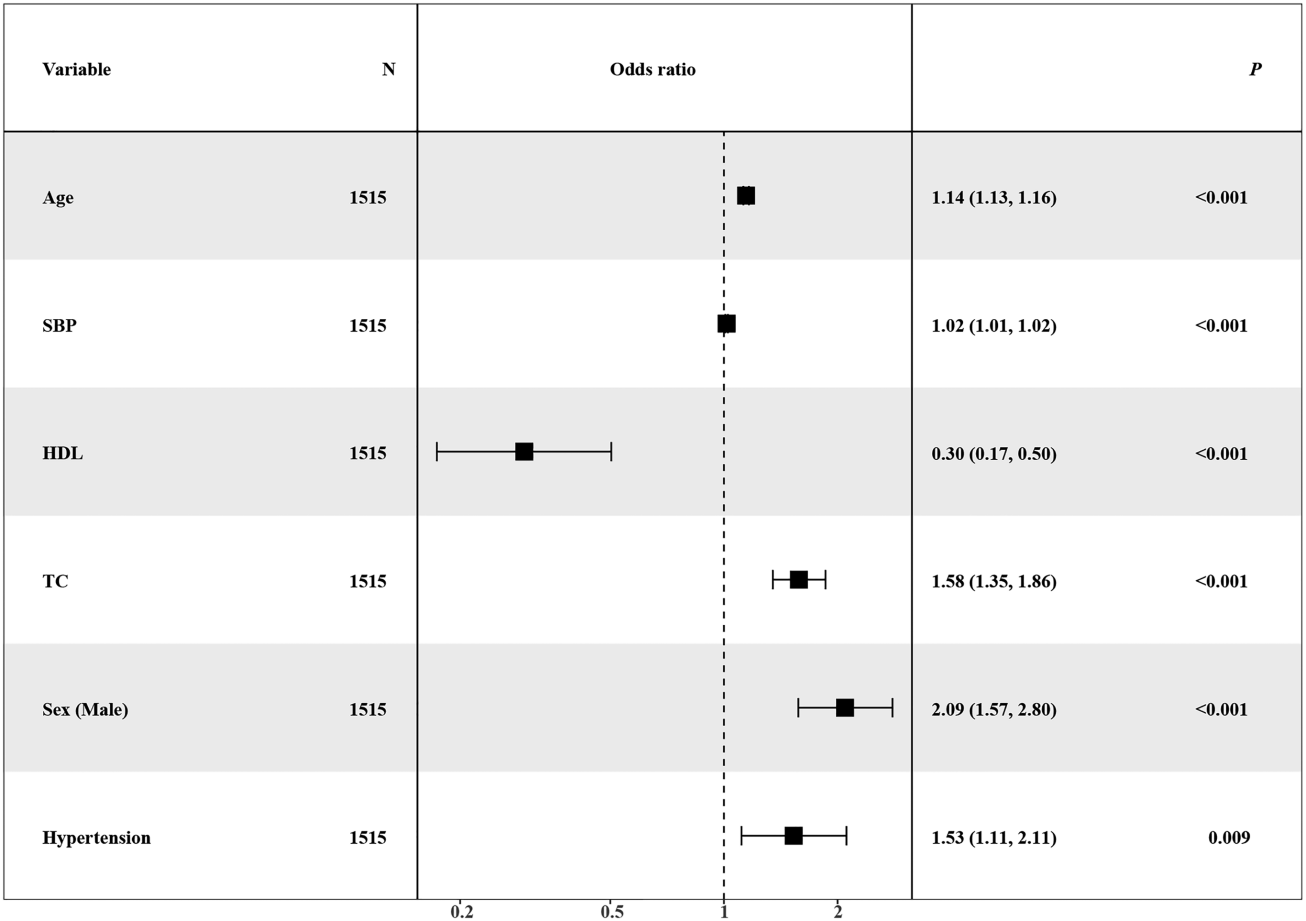
Forest plot showing logistic regression multivariate analysis of CAS.

Simultaneously, the AUC values for each learner on the training set were computed and visually represented in the figure. The outcomes of different machine learning algorithms on the training and test sets were summarized in Table 2. Remarkably high accuracy levels (79%–100%) were achieved by all classifiers during the training phase. However, when applied to the test set, the overall accuracy dropped to 75%. Notably, an accuracy of 82% was achieved by the logistic classifier, outperforming its counterparts. This discrepancy underscores potential challenges in the model's generalization to unseen data, with the logistic regression model exhibiting greater robustness during the transition from training to testing phases. Therefore, the logistic regression model (Figure 3) was selected as the final model, incorporating six variables: age, systolic pressure, hypertension, total cholesterol, HDL cholesterol, and sex. Based on these six variables, the model was named the AP2C2S model. Specifically, age showed a significant positive correlation with CAS (OR = 1.14, 95% CI: 1.13–1.16, *P* < 0.001), while systolic pressure also demonstrated a positive correlation (OR = 1.02, 95% CI: 1.01–1.02, *P* < 0.001). Conversely, high-density lipoprotein (HDL) exhibited a significant negative correlation with CAS (OR = 0.30, 95% CI: 0.17–0.50, *P* < 0.001), indicating its role as a protective factor against CAS. Additionally, total cholesterol (TC) showed a positive correlation with CAS (OR = 1.58, 95% CI: 1.35–1.86, *P* < 0.001). Gender, when treated as a categorical variable, was significantly associated with CAS, with males showing a higher risk (OR = 2.09, 95% CI: 1.57–2.80, *P* < 0.001). Moreover, individuals with a history of hypertension exhibited a significant positive correlation with CAS (OR = 1.53, 95% CI: 1.11–2.11, *P* = 0.009). These findings underscore the significance of these variables in predicting CAS risk and justify their inclusion in the AP2C2S model.

**Table 2:** Comparison of the performance of machine learning classifiers.

Learner	Area under the curve training set	Area under the curve test set	Sensitivity	Specificity	False negative rate	False positive rate
Logistic regression	0.8505827	0.8208542	0.8216663	0.6595230	0.1783337	0.3404770
Support vector machines	0.9395673	0.8061250	0.8365956	0.6162075	0.1634044	0.3837925
XGBoost	1.0000000	0.7876161	0.8043851	0.6269529	0.1956149	0.3730471
Decision trees	0.7886977	0.7631142	0.8412331	0.5975671	0.1587669	0.4024329
Random forests	0.9995162	0.8145973	0.8596306	0.6207752	0.1403694	0.3792248
Scale.oversample.classif.log_reg	0.8495273	0.8218000	0.7595176	0.7214073	0.2404824	0.2785927
Scale.oversample.classif.svm	0.9466890	0.7996170	0.7871304	0.6672630	0.2128696	0.3327370
Scale.oversample.classif.xgboost	1.0000000	0.7943870	0.8020730	0.6502087	0.1979270	0.3497913
Scale.oversample.classif.rpart	0.7904094	0.7518600	0.7676433	0.6703518	0.2323567	0.3296482
Scale.oversample.classif.ranger	0.9996804	0.8163943	0.8366222	0.6718664	0.1633778	0.3281336

This set of machine learning pipelines (“scale.oversample.classif.log_reg”, “scale.oversample.classif.svm”, “scale.oversample.classif.xgboost”, “scale.oversample.classif.rpart”, “scale.oversample.classif.ranger”) corresponds to different classifiers such as logistic regression, support vector machine, XGBoost, recursive partitioning tree (rpart), and Ranger random forest, aimed at accomplishing the final classification task. The ensemble framework is designed to enhance the model's ability to handle imbalanced data, thereby improving the overall performance of the classification algorithm.

**Figure 4:**
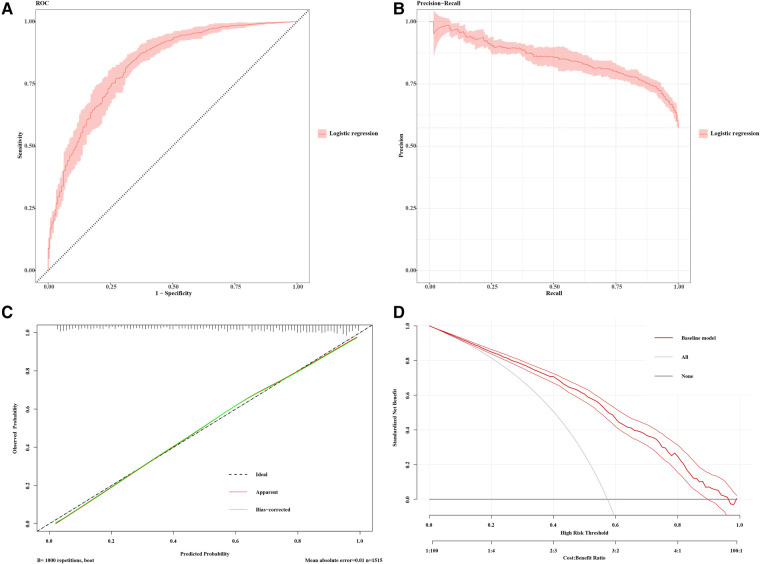
Model performance for CAS prediction. (**A**) Receiver-operating characteristics (ROC) and (**B**) precision recall curve (PRC) plots for the predictive model (AP2C2S model) for discrimination of patients with carotid atherosclerosis in the multiple community populations. The 95% CIs of precision and sensitivity are shown as pink ribbons in the PRC and ROC curves, respectively. (**C**) Calibration curve for the AP2C2S model. The plot shows the agreement between predictions from the model and what was actually observed. (**D**) Decision curve analysis for the AP2C2S model. The net benefit (y-axis) is the net proportion of community populations with CAS who, based on the decision strategy, would correctly be recommended further intervention at the same rate that populations with non-CAS would not be recommended further screening. The threshold probability (x-axis) indicates the range of predicted risk levels above which patients and their physicians might opt for further screening. The gray and black lines (horizontal) represent the scenarios where all or none of the community populations would be prospectively determined by the AP2C2S model, respectively. The red line demonstrates the net benefit of the risk model dependent at the chosen risk threshold. The accompanying thinner lines represent the 95% confidence intervals.

Expanding upon our analysis, we extracted the regression model and generated ROC and PRC curves (Figures 4A,B) to further assess the model. We performed consistency checks using the calibrate function and depicted the calibration curve (Figure 4C). Additionally, employing decision curve analysis allowed us to assess patient benefits at different thresholds, providing insights into the model's potential value in real clinical decision-making (Figure 4D). This series of analyses and evaluations contributes to a thorough understanding of the model's performance and applicability.

### Development of a risk scoring tool (AP2C2S)

Firstly, we categorized the variables of the final model as per the methodology section, assigning values to each variable based on the description provided (refer to Table 3). Subsequently, utilizing the equation of the multiple logistic regression model, we calculated the risk prediction probability for each corresponding score. Through this iterative process, we established a comprehensive table illustrating the correspondence between total scores and risk prediction probabilities, as depicted in Table 4.

**Table 3:** Development of a risk factor scoring tool using multifactor logistic regression.

Factors	Categories	Reference value (*Wij*)	*β*i	*βi* (*Wij−WiREF*)	Pointsij = D/B = (Wij−WiREF) * βi/B
Age			0.127		
	35–44	39.5 = W1REF		0	0
	45–54	49.5		1.27	4
	55–64	59.5		2.54	8
	65–74	69.5		3.81	12
	≥75	78.5		4.953	15
SBP			0.022		
	<120	107		−0.396	−1
	120–129	125 = W2REF		0	0
	130–139	135		0.22	1
	140–149	145		0.44	1
	150–159	155		0.66	2
	≥160	170		0.99	3
HDL			−1.28		
	<1.14	0.99		0.2944	1
	1.14–1.29	1.22 = W3REF		0	0
	1.30–1.50	1.40		−0.2304	−1
	≥1.51	1.89		−0.8576	−3
TC			0.55		
	<4.70	3.98 = W4REF		0	0
	4.70–5.33	5.02		0.572	2
	5.34–6.04	5.69		0.9405	3
	≥6.05	7.23		1.7875	5
Sex			0.88		
	Female	0 = W5REF		0	0
	Male	1		0.88	3
Hypertension			0.39		
	No	0 = W6REF		0	0
	Yes	1		0.39	1

HDL, high-density lipoprotein; SBP, systolic blood pressure; TC, total cholesterol.

Wij (Reference Value of Risk Factor Group): Represents the reference value of the j-th category of the i-th risk factor group. Typically, the median of this category is selected as the reference value. WiREF (Basic Risk Reference Value): Represents the baseline risk reference value of the i-th risk factor. When constructing the scoring tool, an appropriate group is selected as WiREF, with its score set to 0. Other groups receive positive or negative scores based on their relationship with WiREF. Risk Score Calculation: Utilizes the regression coefficients βi estimated by a multiple logistic regression model and the reference values Wij of each risk factor group to calculate the distance D between each group and the baseline WiREF. The formula is as follows: D = (Wij - WiREF) * βi. The score for each risk factor (Pointsij) represents the calculation derived from the multiple logistic regression model, indicating the score of the ith risk factor in the jth category. This score is based on the difference between the reference value (Wij) and the baseline risk reference value (WiREF) of that category, multiplied by the regression coefficient (βi) of the risk factor, and divided by a constant (B). This constant, B, is utilized to convert the units of the regression coefficients into scores, ensuring consistency in the use of scores within the risk scoring system.

**Table 4:** The correspondence table between total score and risk prediction probability.

Point total	Estimate of risk	Point total	Estimate of risk	Point total	Estimate of risk
−1	0.88195759	9	0.452914929	19	0.084023053
0	0.857062087	10	0.39917591	20	0.068567823
1	0.827940365	11	0.347760001	21	0.055782307
2	0.794309407	12	0.299663659	22	0.045264987
3	0.756041843	13	0.25561233	23	0.03665365
4	0.713225204	14	0.216039055	24	0.029629715
5	0.666211583	15	0.18110187	25	0.023918357
6	0.615644079	16	0.150728336	26	0.019286027
7	0.56244723	17	0.124673352	27	0.015536572
8	0.507774373	18	0.102578251	28	0.012506764

The seamless integration of this grouping and calculation procedure facilitates a systematic evaluation of each variable's contribution to the final model. It ensures a thorough and precise prediction of risk probability for patients. This methodical approach underscores the scientific rigor and practical utility of our risk assessment model in a clinical context.

To illustrate the application of the AP2C2S risk scoring tool concretely, we provide an example based on Tables 3, 4. Let's consider a 55-year-old male patient with a systolic blood pressure of 135 mmHg, total cholesterol (TC) of 5.5 mmol/L, high-density lipoprotein (HDL) of 1.2 mmol/L, and a history of hypertension. Referring to Table 3, we can assign points for each risk factor: Age (55–64 years): 8 points; Gender (male): 3 points; SBP (130–139 mmHg): 1 point; HDL (1.14–1.29 mmol/L): 0 points; TC (reference range): 3 points; Hypertension (yes): 1 point. Adding up these points, the patient's baseline total score is 16 points. Next, referring to Table 4, we find the corresponding CAS risk prediction probability based on the total score. In this example, a score of 16 corresponds to a risk prediction probability of 15.07%.

## Discussion

Our study revealed a 57.4% prevalence of CAS among 1515 participants, underscoring a significant risk, especially in the middle-aged and elderly population. CAS patients exhibited higher age and positive correlations with traditional cardiovascular risk factors, including systolic blood pressure, age, history of hypertension, male gender, and total cholesterol. Notably, we identified high-density lipoprotein (HDL) as a protective factor against CAS, highlighting its role in risk mitigation. These findings indicate that total cholesterol and HDL levels could serve as significant predictors of CAS in community-dwelling men. This underscores the importance of managing cholesterol and increasing HDL levels to prevent the development of atherosclerosis. Specifically for elderly men with a history of hypertension, early adoption of secondary cardiovascular prevention measures is strongly recommended. Furthermore, leveraging advanced machine learning techniques, such as SVM, XGBoost, decision trees, random forests, and logistic regression, we achieved high accuracy during training (79%–100%). The logistic classifier, with an 82% accuracy, exhibited superior robustness in transitioning from training to testing, leading to its selection as the final model. This comprehensive approach enhances our understanding of CAS while providing a practical tool for risk assessment and personalized preventive strategies.

A traditional belief was that HDL particles, known for their role in reverse cholesterol transport, conferred cardiovascular benefits. However, current insights into HDL highlight its role in suppressing inflammation, oxidative stress, and stimulating endothelial function (11). Additionally, animal studies have indicated that recombinant HDL may inhibit the expression of carotid VCAM-1 (12) or aortic VCAM-1 and ICAM-1 (13). HDL has also been demonstrated to reduce the surface expression of ICAM-1, VCAM-1, and E-selectin by activating annexin A1 (14), a recognized anti-inflammatory mediator. In summary, the collective evidence from experimental models suggests that HDL particles directly influence endothelial cells, inhibiting proteins associated with endothelial activation. This mechanism holds the potential to decelerate the progression of atherosclerosis.

Notably, this study integrates traditional epidemiological analysis with advanced machine learning techniques, culminating in the creation of a practical risk scoring tool. By initially recruiting a substantial sample and utilizing carotid ultrasound to identify atherosclerosis, the research delves into population characteristics, revealing significant associations with demographic factors. The application of machine learning algorithms enhances the study's predictive capabilities, with logistic regression emerging as the most robust model (15). The incorporation of ROC and PRC curves, calibration checks, and decision curve analysis ensures a thorough evaluation of the model's performance and clinical utility. Additionally, the development of a risk scoring tool (AP2C2S) based on logistic regression results contributes to a systematic and precise assessment of individual risk probabilities. This holistic methodology reflects a novel and integrated approach to investigating and managing CAS, offering valuable insights for both research and clinical applications.

While our study yields valuable insights, certain limitations need consideration. The cross-sectional nature impedes causal inference, necessitating future longitudinal investigations. Additionally, the study's regional focus may impact generalizability, prompting caution in extending findings to broader populations. It is also worth noting that the AP2C2S model has not undergone direct comparison with the Systematic Coronary Risk Evaluation (SCORE) system (16) or other currently utilized scoring systems for carotid artery disease risk assessment. Therefore, while our findings provide valuable insights into the risk factors associated with carotid atherosclerosis, further research is needed to compare the AP2C2S model with other scoring systems and to validate its predictive accuracy across different demographic groups.

The risk scoring tool developed in this study serves as a foundation for further refinement and validation in diverse populations. Future research could explore additional biomarkers or imaging modalities to enhance risk prediction accuracy. The study sets the stage for evaluating personalized interventions based on risk scores, contributing to targeted and efficient preventive strategies.

Given the prolonged progression of atherosclerosis, our study's “preventive strategies” aim to achieve two key objectives: primary prevention for identifying high-risk individuals susceptible to cerebrovascular and cardiovascular complications, and secondary prevention targeting existing CAS patients. For primary prevention, our AP2C2S model facilitates the identification of candidates who could benefit from lifestyle modifications and early medical interventions, thereby reducing the risk of stroke. In the realm of secondary prevention, the same model serves as a valuable tool for prioritizing patients requiring heightened surveillance and more aggressive treatment approaches to impede CAS progression.

## Conclusions

In conclusion, our study unveils a significant prevalence of CAS within the community, especially among the elderly. The introduction of the AP2C2S risk scoring tool, validated through the logistic classifier's robust performance across training and testing phases, offers a refined approach to risk assessment. This tool holds promise for identifying high-risk individuals within community health initiatives, potentially streamlining the process of screening and clinical intervention. By emphasizing the critical role of cholesterol management, particularly high-density lipoprotein (HDL), our research provides actionable insights that could inform CAS prevention strategies. However, we recognize the imperative for rigorous and extensive validation to ensure the tool's practicality and effectiveness in diverse real-world settings.

## Data Availability

The original contributions presented in the study are included in the article/Supplementary Material, further inquiries can be directed to the corresponding author.
